# Perceived barriers to and facilitators of physical activity, using the COM-B model for behavioural change, in people with chronic pain: a qualitative evaluation of patient and stakeholder perspectives

**DOI:** 10.1186/s12889-025-25252-0

**Published:** 2025-11-07

**Authors:** Cassie Higgins, PM Dall, C. Leese, P. Adair, PA Cameron, SK Inglis, A. Christogianni, BH Smith, L. Colvin

**Affiliations:** 1 Division of Population Health and Genomics, School of Medicine, University of Dundee, Dundee, UK; 2 Research Centre for Health, School of Health & Life Sciences, Glasgow Caledonian University, Glasgow, UK; 3 School of Psychology, Queen’s University Belfast, Belfast, UK

**Keywords:** Chronic pain, Physical activity, Barriers, Facilitators, Qualitative analysis, Framework analysis

## Abstract

**Background:**

Physical inactivity is a substantial public health concern and challenging problem to address. Physical activity (PA) is shown to be of therapeutic benefit to those living with chronic pain (CP), but intervention studies have often been unsuccessful in promoting sustainable engagement with PA in this population. To develop a more comprehensive understanding of the barriers to and facilitators of sustainable PA in people with CP and to frame the findings within the six COM-B constructs.

**Method:**

This qualitative study was informed by behaviour change theory (the COM-B model), and participants were drawn from two settings. First, 36 adults living with moderate-severe CP were recruited from a specialist NHS pain service (‘patients’). Secondly, 15 stakeholders were recruited to represent a range of healthcare sectors involved in supporting people with CP. One-to-one semi-structured interviews, informed by the COM-B model, were conducted between May 2020 and October 2021. Interviews were conducted by telephone or using online conferencing software and were transcribed *verbatim* and anonymised. The Framework Method was used to analyse the data. First, two independent researchers conducted line-by-line coding and, using a consensus approach, collapsed the findings to produce subthemes. Secondly, these inductively identified subthemes were mapped to predetermined themes (i.e. the six COM-B constructs) using a consensus approach involving three researchers.

**Results:**

Many patients reported prohibitive pain intensity, worsening symptoms and lack of motivation as barriers to physical activity and that effective pain management, personalised support and social interaction facilitated physical activity. The inclusion of stakeholder perspectives was valuable in developing a more comprehensive understanding of the barriers to PA encountered by people with CP and also of the facilitators that are successful in promoting sustainable PA in this population. For example, stakeholders highlighted additional barriers, such as people’s limited financial resources and their perceptions of physical activity being counterintuitive when experiencing pain.

**Conclusions:**

The barriers to and facilitators of PA in people with CP are numerous and complex, and stakeholder perspectives can broaden this understanding. Barriers and facilitators were successfully mapped to the COM-B constructs in a way that could inform future PA intervention design.

**Supplementary Information:**

The online version contains supplementary material available at 10.1186/s12889-025-25252-0.

## Background

For more than a decade, physical inactivity has been widely recognised as one of the greatest public health concerns of the 21 st century [1–4], and studies show that this is a particular issue in people living with chronic pain (CP) [5–7]. Physical inactivity is generally considered to be activity levels that falls below those recommended in current guidelines. Advice from the World Health Organization [8] and the UK’s Chief Medical Officers [9] recommends at least 150 min per week of moderate activity or at least 75 min of vigorous activity. In people with CP, physical inactivity is shown to increase risk of mortality [10], while physical activity (PA) is shown to reduce pain severity/sensitivity [11, 12], increase pain tolerance [13] and improve quality of life [11, 14, 15]. Whilst the health benefits of PA to people with CP are well-documented, interventions designed to increase PA in people with CP who lead relatively sedentary lifestyles are frequently ineffective. Indeed, several recent systematic reviews have shown no effect of interventions at increasing sustainable PA in this population [16–18]. In consequence, there is an urgent need to develop a comprehensive understanding of the barriers and facilitators to PA in people with CP, particularly within the context of a behavioural change model to better inform effective interventions.

Over the past two decades, in response to a growing recognition of the importance of PA in the management of chronic, painful conditions, qualitative studies have attempted to develop an understanding of the barriers to engaging with PA and the facilitators that might support such engagement [19–24]. Most qualitative studies have focused solely on patient perceptions; however, there is demonstrated value in also including stakeholder perspectives [25], such as those of healthcare professionals, PA trainers and third sector care staff, and in interpreting the data within a model of behavioural change, such as the COM-B model, to maximise the potential of developing effective interventions [26].

**Fig. 1:**
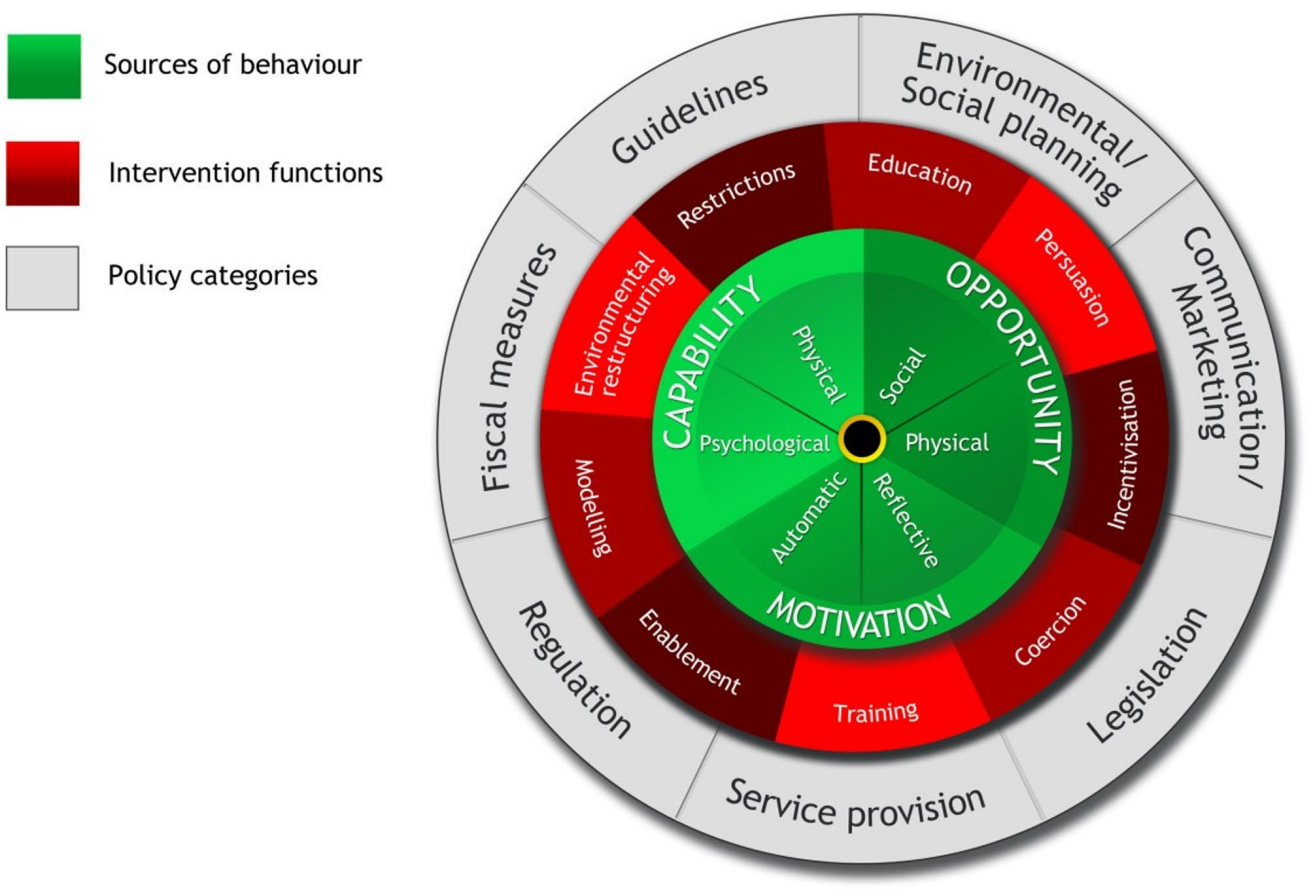
Behaviour Change Wheel (BCW) from the COM-B model for behavioural change [Reproduced from Michie et al. (2011), under the Creative Commons Attribution License (CC BY 2.0): https://creativecommons.org/licenses/by/2.0/.]

The COM-B Model for Behavioural Change [27] is centred on a system of behaviour and is extended to capture the range of mechanisms that could be involved in change, both internal (psychological and physical factors) and external (environmental factors). At the core of the model (the behaviour system), they propose that an individual must have the capability (C), opportunity (O) and motivation (M) in order to achieve a behaviour (B) and that one or more of these constructs must change in order to achieve behavioural change. They acknowledge that these factors interact over time; therefore, behaviour is considered a dynamic system with positive and negative feedback loops. The authors provide a visual representation of their model, referred to as the ‘Behaviour Change Wheel’ (BCW [27]; see Fig. 1), around which are positioned the nine intervention functions aimed at addressing deficits in one or more of these conditions and then seven categories of policy that could enable those interventions to occur. The BCW has been successful in characterising national interventions (e.g. the English Department of Health’s 2010 tobacco control strategy and the National Institute of Health and Clinical Excellence’s guidance on reducing obesity) [27].

The present study set out to develop a more comprehensive understanding of the barriers to and facilitators of sustainable physical activity in people with chronic pain and to frame the findings within the six COM-B constructs. Achieving this aim could facilitate opportunities for the development of relatively personalised interventions aimed at effectively promoting sustainable PA in people with CP using the COM-B model to affect behaviour change.

## Methods

The study design was qualitative, informed by behaviour change theory (the COM-B model), and participants were drawn from two settings. First, 36 adults living with CP (hereafter termed ‘patients’) were recruited from a specialist National Health Service (NHS) pain service in Scotland. NHS healthcare is available to all in the country and is free at the point of contact, and access to specialist pain services requires a referral from a general practitioner (GP), secondary care clinician or other appropriately qualified healthcare professional. A specialist setting was selected to ensure the recruitment of patients with a range of severe and debilitating painful conditions, such as fibromyalgia, osteoarthritis and chronic back pain. Patient inclusion criteria were: (1) at least 18 years of age; (2) consented to being contacted for research purposes and able to give informed consent for study participation; (3) referred to the Pain Service; (4) pain that had persisted for at least 6 months; (5) moderate-severe pain, assessed prior to study inception using a 0–10 numeric rating scale (employing the commonly-used cut-point of >3 [28]); and (6) no clinical contraindications to wearing an accelerometer. Patients were excluded if they were in active treatment for cancer or were participating in the clinical phase of an interventional study or had done so within the past 30 days. Pain score (obtained from the numeric rating scale) and PA level (number of steps per day, obtained from ActivPAL accelerometers, which were worn by all patients) are described for each patient participant.

Secondly, 15 stakeholders were recruited nationally to represent a range of healthcare sectors, including GPs, physiotherapists and other service providers involved in supporting patients with CP. Stakeholder inclusion criteria were: (1) at least 18 years of age; (2) able to give informed consent for participation; and (3) considered to be fulfilling a relevant stakeholder role (i.e. GPs and allied health professionals (AHP)s, nurses, pharmacists, carers, staff at third sector organisations, leisure centre staff and others identified through the study team working in conjunction with the Green Health Partnership, Pain Concern and Versus Arthritis). Stakeholders were recruited to provide an alternative insight into the barriers and facilitators encountered by patients living with CP, which was triangulated with the data provided by patients.

Throughout the manuscript, where referring to patient participants the term ‘patients’ is used and, where referring to stakeholder participants, the term ‘stakeholders’ is used. The term ‘participants’ is used to refer to participants from both groups.

One-to-one semi-structured interviews were conducted with participants between May 2020 and October 2021, during the height of the COVID-19 pandemic, and written consent was taken from all participants prior to participation. Interviews were conducted using online conferencing software (Microsoft Teams) or by telephone, due to imposed lockdown restrictions, and the interview topic guides for the patient participants (Table S1) and stakeholder participants (Table S2) were based on the COM-B model [27]. Interviews were recorded using either an Olympus audio recorder (telephone interviews) or using video conference software (Microsoft Teams), transcribed verbatim and anonymised prior to data analysis.

Using an inductive-deductive approach, the Framework Method [29] was employed in interpreting the data produced in the present study, and data analysis was conducted using NVivo 12 Plus [30]. Data were analysed separately for the patient and stakeholder participant groups. First, inductive line-by-line coding was performed independently by two researchers using three patient transcripts (CH & PA) and three stakeholder transcripts (CH & AC). Secondly, using a consensus approach, these codes were collapsed to identify a preliminary list of subthemes – the initial iteration of the thematic framework. The thematic framework was considered a ‘living document’, which was not finalised until the final transcript had been analysed and all possible subthemes had been identified. Thirdly, the inductively identified subthemes were mapped to the predetermined themes (i.e. the six COM-B constructs). This final step in the process was conducted using a consensus approach involving three researchers (CH, CL & BHS) who met three times within a two-week period, with opportunities for input from all team members to review the mapping. A consensus approach was used to minimise the bias that might have been introduced had only one person conducted the mapping process, and the consensus discussions were undertaken intensively, rather than over a protracted period, to maximise consistency in the mapping process.

Further information about this study can be found on ISRCTN (registration number: ISRCTN95480359) [31].

## Results

A total of 36 patients and 15 stakeholders participated in interviews, and their characteristics are shown in Table 1. The patient cohort comprised more females (61%; *n* = 22) than males (39%; *n* = 14), and ages ranged from 21 to 81 years (mean = 52 ± 16 years). The mean pain intensity score was 5.03 ± 1.14 on a 0–10 visual analogue scale: 3 (9%) patients reported experiencing ‘mild’ pain (scoring ≤ 3); 27 (77%) reported ‘moderate’ pain (scoring 4–6); and 5 (14%) reported ‘severe’ pain (scoring ≥ 7). Number of steps per day, based on accelerometer data, ranged from 702 to 23,945 (mean = 6,881 ± 5,106).

**Table 1:** Patient and stakeholder demographic characteristics and measures of pain and physical activity in patient participants

Patient characteristics							Stakeholder characteristics			
ID		Gender	Age	Physical activity (steps per day) *	Pain score (0–10 VAS)		ID		Gender	Role working with people with pain
A02		Male	43	4,606	3		S01		Male	PA Project Manager
A05		Female	51	5,920	5		S02		Female	Physiotherapist
A06		Female	67	9,798	6		S03		Female	Social prescriber
A07		Female	43	11,247	NR		S04		Female	General practitioner
A08		Male	33	1,146	6		S05		Female	Service policy maker
A09		Female	72	4,088	7		S06		Male	Pharmacist
A10		Male	67	7,410	5		S07		Female	General practitioner
A11		Female	51	9,340	6		S08		Female	Pharmacist
A12		Female	48	3,057	7		S09		Female	PA Project Manger
A13		Female	21	12,712	6		S10		Male	Psychologist
A14		Male	57	20,252	5		S11		Female	Pain support worker
A16		Male	76	7,153	4		S12		Male	GP trainee
A18		Female	22	4,149	4		S13		Male	Pharmacist
A19		Male	64	12,568	7		S14		Female	CP nurse specialist
A20		Female	39	3,555	3		S15		Male	Leisure centre manager
A21		Female	73	3,924	7					
A22		Male	53	7,155	5					
A23		Female	25	3,077	5					
A24		Female	46	7,519	4					
A25		Female	79	7,063	4					
F01		Female	40	702	7					
F02		Male	54	10,207	2					
F06		Male	38	1,527	5					
F07		Female	59	5,947	5					
F08		Female	53	4,784	5					
F09		Female	67	1,204	5					
F10		Male	66	5,144	5					
F11		Female	52	8,134	5					
F12		Male	61	23,945	6					
F13		Female	58	2,579	5					
F14		Male	39	2,174	5					
F15		Female	22	11,002	5					
F17		Female	31	9,286	6					
F18		Male	51	6,982	6					
F19		Female	52	1,493	6					
F21		Male	81	NR	5					

* Measured using the ActivPAL accelerometer, and, to contextualise, guidance suggests 4,500 to 5,500 steps per day for improved health related quality of life, > 7,000 steps per day for better immune function, and 8,000 to 10,000 steps per day for an effect on metabolic syndrome and maintenance of weight (Scottish Government, 2019)

*NR* no response

Table 2 shows the subthemes that were identified during participant interviews, grouped by theme (i.e. COM-B constructs), by participant group (patients and stakeholders) and by facilitators/barriers. Each of the subthemes was mapped to one theme (i.e. the six COM-B constructs).

**Table 2:** Subthemes identified during participant interviews, grouped by theme (COM-B constructs), by participant group (patients and stakeholders) and by facilitators/barriers

Themes	Subthemes			
	Barriers to PA		Facilitators of PA	
	Patient-reported barriers	Stakeholder-reported barriers	Patient-reported facilitators	Stakeholder-reported facilitators
Physical capability	• PA limited by clinical symptoms (primarily pain problems) • PA limited by tiredness and lack of energy • Medication side effects • PA worsens symptoms • Being overweight makes PA difficult • Confined to wheelchair	• Difficult to rebuild fitness post-lockdown • Reasonable fitness level required for participating in group activities	• Effective pain management • Increasing confidence with PA facilitates PA • Self-identifying PA that works with personal limits • Rescue plan in place in case of nor coping with PA • Experience that PA improves pain symptoms • Experience of building PA slowly and increasing fitness	• Feeling the benefit of PA • PA suited to personal limits
Psychological capability	• Lack of knowledge about PA • Mentally exhausted by thinking about PA • PA limited by affective disorders	• Affective issues limit PA • Inaccurate perceptions of what constitutes PA • Lack of guidance on safe PA for people with chronic pain • Lack of patient knowledge about PA • Lack of research evidence regarding the benefits of PA • Lack of stakeholder knowledge about PA and how to access PA • Lack of patient knowledge about changing guidelines • Lack of stakeholder knowledge about who takes responsibility for promoting PA	None	• Guideline to promote PA encourages healthcare professionals to promote PA • Helping patients understand that PA doesn’t cause harm • Time for patients to change their thinking about engaging with PA • Patients need to understand why PA is beneficial
Automatic motivation	• Fear of judgement of others • Fear of trying new things • Fear of physical effects of PA • Fearful and lacking confidence outside the home • Overwhelmed by instruction to exercise every day • Lack of desire and motivation to engage with PA • Some PA terminology is off-putting • Fear of catching COVID • Fearful for safety at night	• Experiencing anxiety when trying PA • Fear of being unable to do PA • Fear of catching COVID • Fear of causing harm to self • Fear of judgement of others • Fear of trying new things • Fear of worsening pain • Lack of motivation or desire to engage with PA • Lack of patient engagement • Lack of weight loss causes disillusionment with PA • Overdoing exercise and causing discomfort and demotivation	• Sense of achievement • Desire for weight loss • Desire to engage with work • Desire to get out of the house • PA needs to have substantial impact on pain to be worthwhile • Desire and willingness to engage with PA • Confident exercising in local area	• PA needs to be enjoyable • Rapid positive outcomes from PA motivate further PA • Role models motivate PA
Reflective motivation	• Clinical symptoms (primarily pain problems) • Unpredictable nature of pain limits commitments to do things • Tiredness and lack of motivation	• Experiencing that PA worsens pain and discourages further PA • Lack of belief in the benefits of PA • PA is counterintuitive when in pain • Some conditions don’t respond positively to PA	• Financial commitment to PA • Health and wellbeing benefits other than pain management • PA needs to be enjoyable and novel • Planning PA facilitates PA • Pushes self to achieve PA • Reminder from activity tracker • Watching PA videos	• Aiming for weight loss or avoidance of weight gain for patients • Belief that medicalisation of PA encourages compliance • Belief that PA improves mobility for patients • Belief that PA improves pain symptoms for patients • Belief that PA increases energy levels • Belief that PA is a distraction from pain for patients • Belief that pain education is more important than PA promotion in encouraging PA • Belief that patient should aim for a normal life • Expectation that building muscle tone helps prevent falls • Expectation that building PA slowly and setting small goals will encourage PA • Expectation that PA improves mental health for patients • Identifying PA that avoids exacerbating pain • Keeping a diary to document progress • Need for strategies other than analgesics for pain management • Restoring movements that patients would like to have • Use of visualisation and emulation to encourage movement
Physical opportunity	• COVID lock-down restricted access to facilities • Lack of appropriate footwear • Lack of suitable PA facilities • Lack of transport • Lack of time • Weather limits PA	• Confinement in prison limits opportunities for PA • COVID has increased waiting times, limited healthcare and prevented PA engagement • COVID has limited access to PA opportunities • Lack of access to suitable PA facilities • Lack of financial resources • Lack of integrated healthcare limits access to PA facilities • Lack of resources for one-to-one support • Lack of services and PA facilities in rural areas • Lack of time to engage with PA • Lack of transport to access PA facilities • Short-term interventions don’t foster sustainability • Waiting lists for medical and support services prevent PA engagement	• Safety features in environment • Dog walking facilitates PA • Convenient or ergonomic household and gardening devices • Engaging with domestic chores • Easy and convenient access to PA facilities • PA through physically active job • Physically active hobbies and interests	• Comfortable surroundings in PA environment • Easy access to PA • Extra time for professionals to meet patient needs and support PA engagement • Family-friendly PA supports those with no childcare • More frequent medical reviews to explore alternatives to long-term prescribing • Online PA sessions • PA opportunities that fit around other people’s commitments • Warm water-based PA makes PA easier
Social opportunity	• Dislike of PA without company • Family commitments	• Bad advice from health professionals causing harm and fear of trying PA again • Gaps in service to support PA engagement • Lack of available education about acute vs. chronic pain • Lack of personalised PA engagement • Lack of sensitivity and understanding from health professionals • Lack of support from specialist pain services for patients and healthcare professionals • Lack of time for professionals to engage with patients • PA activities not the social norm for some patients • PA is not always promoted by healthcare systems • Patients have their benefits stopped due to engaging in PA • Professionals don’t explain the benefits of PA • Stigmatising beliefs held by healthcare professionals • Terminology can be demotivating	• Commitment to others • Company of people who could empathise • Playing with children • Exercising with company • Positive effect from helping others • Prefers to exercise on own or in own space • Support and understanding from friends or family • Support from health and wellbeing professionals	• Collaboration between agencies supports PA engagement • Company of people in similar circumstances • COVID has increased PA through focus on health and use of time • Demedicalisation of PA reduces stigma • Developing trusting relationships with healthcare professionals • Education about the benefits of PA encourages PA • Empowering people to want to engage with PA • Increased fitness affords opportunities for greater community participation • Legislative action to promote PA • Making a commitment to others • PA with company • Personalised PA recommendations • Promoting ideas about new activities and planning routes into them • Reducing pressure to engage with PA may facilitate engagement with PA • Support and understanding from health and wellbeing professionals • Support from community pharmacies could help to promote PA

An overview of the prominent barriers and facilitators to PA identified by people with CP and stakeholders is presented in the remainder of this section, organised by the COM-B constructs. The subsections relating to each of the COM-B constructs is further divided into barriers and facilitators. Quotes from patients are coded either ‘Axx’ or ‘Fxx’, and quotes from stakeholders are coded ‘Sxx’.

### Capability – physical

#### Barriers

Patients reported that PA was limited by physical symptoms, primarily prohibitive pain intensity, and that the pain experience worsened when performing PA:

*I used to love taking the dog out for a walk and now I just can’t do it*,* I can’t even walk down to the bottom of the road where the paper shop is because it’s just too painful [A12]*

*… whenever I do the garden*,* I know when I’m doing it and I know the next day I’m going to be in horrific pain and it’s going to be a really bad day [A11]*

Patients also felt that the unpredictable nature of pain prevented them from committing to participating in activities because failing to honour commitments impacted either on them or on others:

*Planning is very*,* very difficult*,* it’s made a massive inroads into both myself and my wife’s lives*,* it’s very difficult to plan anything with any certainty at all [A16]*

#### Facilitators

Patients reported that effective pain management was an important facilitator for engaging with PA:

*I just needed something to give me that wee bit of pain relief so I could cope in the mornings and do stuff*,* get up*,* get a shower [F08]*

and many felt that PA could achieve this effectively:

*I find walking helps as well. If I go out on slow walks it just helps the pain if I do that but then it is painful as I’m walking but the pain seems to ease as I’m walking [A09]*

Additionally, for some, having a rescue plan in place facilitated PA engagement:

*It’s like I’ve got a wheelchair if need be and so I’d give it a go and would take that as a back up [A08]*

### Capability – psychological

#### Barriers

Both groups felt that affective symptoms limited PA:

*I’ve got lack of motivation anyway because of depression [A12]*

*Mental health, that is a massive barrier [S02]*

and stakeholders highlighted the dynamic relationship between pain and mental health, which further limits opportunities for PA:

*A lot of people haven’t been out of the house for so long due to their pain they almost develop a secondary social condition where they just become really sort of agoraphobic [S11* *]*

However, the most prominent issue was lack of patient and stakeholder knowledge about the benefits of PA and how to access PA opportunities:

*I would be able to do things but with no knowledge to be able to do more exercise I can’t do it. [F17]*

*I probably wouldn’t recommend anybody going to the gym and that’s probably around my lack of knowledge [S06]*

#### Facilitators

Stakeholders observed that the availability of a PA guideline encouraged healthcare professionals to promote PA to patients but that patients need time to make mental shifts in how they think about PA. They also observed that patients need to understand why PA is beneficial and that PA does not cause harm in people with CP:

*[Exercise is] more beneficial when you know why you’re doing it … [and understand that] this movement isn’t going to cause me any damage*,* but it will take time [S02]*

### Motivation – automatic

#### Barriers

First, both groups reported fear of the physical effects of PA, largely worsening pain or causing harm, as a prominent barrier:

*Sometimes I have concerns that the exercise I’m doing*,* although I pace myself*,* might be doing damage. [A14]*

*If you do something that hurts*,* part of the frustration is the pain at the time*,* and part of the worry is how bad is it going to be in 24 hours’ time or 40 hours’ time? [S12]*

Secondly, participants in both groups reported a lack of patient desire and motivation to engage with PA:

*The actual motivation to go and do it*,* even though it’s only going to maybe take a couple of minutes*,* the actual motivation to go out and do it*,* no. [A12]*

*I think it takes a lot more motivation for someone with chronic pain to exercise than someone that doesn’t have chronic pain. [S12]*

Thirdly, fear of the judgement of others was seen as a substantial barrier by both groups, with a focus on physical signs of health conditions (such as a hernia or skin condition) and lack of capacity for PA:

*There is exercise class but they do a lot of things I couldn’t do. Somebody said to me to sit in a chair and do some of them but without anybody else sitting in a chair and doing exercises I would feel out of place or looked at or something. [A06]*

#### Facilitators

Three prominent facilitators were identified by patients: desire and willingness to engage with PA; desire for weight loss; and desire to get out of the house. Stakeholders failed to recognise desire and willingness in patients; however, it was widely expressed by patients:

*So if there’s something that is clinically proven and something that’s worth trying*,* I would definitely give it a try*,* whatever form of exercise that may be. So I’m open to that. Absolutely. [F06]*

### Motivation – reflective

#### Barriers

Patients reported one prominent barrier: lack of trust in PA guidance:

*There’s been some days I’ve thought whoever thought that up [PA improves pain] must have been off their head because it’s not true [A05]*

and stakeholders reported one prominent barrier: the experience that PA worsens patients’ pain and discourages further PA:

*… psychologists often feel that if we’re going to encourage [PA] that are we at risk of causing further worsening chronic pain. [S10]*

*… they try and do some exercise and it will be sore so that puts them off persevering with that. [S08]*

#### Facilitators

The most prominent facilitator reported by patients was benefits to physical and mental health, and the stakeholders focused specifically on the benefits to mental wellbeing:

*I know that the exercise is necessary to avoid diabetes and muscle issues and all of the other stuff. [F02]*

*There is obviously the strength*,* the fitness but also the feel-good hormones and chemicals that are produced in the brain that help us feel better [A07]*

*… the [physical health] benefits that you’re going to get are almost secondary to the fact that you’re able to get out there for your mental health. [S01]*

An additional prominent factor reported by patients was that PA needs to be enjoyable and novel:

*And what works for me because I don’t want to push myself*,* I want to actually enjoy the exercise to get myself back into it. [F15]*

Additionally, stakeholders believed that building PA slowly and setting small goals would encourage PA:

*It working with the patient to help them find a level that is a baseline and work their way up that’s going to be beneficial and not knock them back even further than they were before. [S10]*

### Opportunity – physical

#### Barriers

The most prominent barrier reported in both groups was restricted access to PA opportunities due to COVID-19 lockdowns, which is likely to have been a transient situation, but they also reported a lack of PA facilities more generally. Patients also reported the impact of bad weather and a lack of time, and stakeholders highlighted lack of financial resources for patients:

*Weather is a demotivating or a motivating factor. [A25]*

*Yes*,* time because it’s obviously half an hour there*,* half an hour back which is a really big chunk of time that I could be trying to get through tasks on my to do list. [A07]*

*… if you Google it and see there’s nothing there then what’s the point in saying you know why don’t you go to so and so. They can only be done during the day and I work so.*

*The cost, there’s a lot of things out there with exercise that’s quite expensive. [S03]*

#### Facilitators

Many of the facilitators identified by patients related to chance opportunities, such as having a physically-active job, engaging with domestic chores or dog-walking activities. They also reported that having easy and convenient access to PA facilities encouraged PA:

*So I thought if I go to a gym*,* there’s one that’s not far from me*,* if I’m tired*,* I could get a bus there*,* you know what I mean? I’ve got some way to get there and get home. [F08]*

Stakeholders suggested a number of additional potential facilitators that were not reported by patients, including making use of online PA classes and the provision of family-friendly PA to support those with no childcare:

*One of the things that we’ve noticed now we’ve got some family classes now where if somebody’s just had a kid or they’ve got a couple of children … they [children] could come in and take part in the classes or do something at the same time. [S01]*

### Opportunity – social

#### Barriers

Patients reported two barriers: dislike of PA without company (lack of enjoyment, lack of confidence and lack of motivation to persevere); and family commitments limiting opportunities for PA.

*It is something that I would be interested in*,* but as I say I’m just not confident to go myself. [A12]*

*Sometimes when the kids are being really difficult and just want to fight with each other all the time and I feel like I can’t just go out and do the garden … I need to be in the same room as them to make sure they’re not at each others’ throats [A07]*

Stakeholders identified numerous additional barriers that related to both issues for patients (PA activities not the social norm for some patients; patients having their benefits stopped due to engaging in PA; and the demotivating effect of some terminology) and concerns around the limitations of service provision (gaps in provision and unhelpful attitudes or stigmatising beliefs held by professionals):

*[Patients] struggle a lot to be validated and believed … so if you go and say something so crass as go exercise*,* all their fears have been confirmed in that one statement*,* you have no idea what is going on with me because if you did you would never have said that in the first place. [S11]*

*I think in health care in particular, it's a lot of judgement. [S05]*

#### Facilitators

The most prominent facilitators identified by patients were support from friends and family and support from health professionals, with stakeholders agreeing with the latter. The other prominent facilitators, identified by both groups, concerned PA with company and, especially, PA in the company of people who could empathise with their difficulties. Stakeholders proposed additional potential facilitators that concerned both service development (enhanced collaboration between agencies and community pharmacy involvement in promoting PA engagement) and the direct patient experience (largely characterised by the provision of education and support to encourage sustainable PA and the way in which professionals engage with patients):

*That’s sometimes where building a relationship with the patient helps*,* though*,* because once they trust you and they kind of know that you’re looking out for them to push it a bit more and say*,* what have you thought about x*,* y and z? … You can get the confidence to have those kind of conversations just because you know the person better. [S13]*

## Discussion

### Summary

The aim of the study was to develop a more comprehensive understanding of the barriers to and facilitators of sustainable PA in people with CP and to frame the findings within the six COM-B constructs. A comprehensive inventory of barriers and facilitators was identified, and each was successfully mapped to one of the COM-B constructs, which could facilitate opportunities for the development of relatively personalised interventions aimed at effectively promoting sustainable PA in people with CP using the COM-B model to affect behaviour change.

Many of the barriers and facilitators that were identified by both the stakeholder group and the patient group echoed previously reported findings, providing further evidence of their importance and validity. However, the patient group raised additional barriers not already addressed in the existing literature that highlight a more complex range of barriers relating specifically to physical capability and automatic motivation. Based on their clinical experience, stakeholders raised an inventory of barriers that had not been raised by patients, spanning reflective motivation, physical opportunity and social opportunity. Regarding social opportunity, stakeholders identified additional barriers that related to both issues for patients and concerns around the limitations of service provision. The stakeholders also observed a range of facilitators, within the domains of psychological capability, reflective motivation, physical opportunity and social opportunity, that had already proven beneficial for some of their patients. Regarding social opportunity, they proposed facilitators that concerned both service development and the direct patient experience.

### Comparison with existing literature

There is a considerable literature examining the barriers to and facilitators of PA in people with chronic, painful conditions; however, there is only a limited literature that frames these facilitators and barriers within a robust framework that could be used to affect behavioural change. A recent rapid umbrella review [26] included 12 systematic reviews that framed barriers and facilitators within the COM-B model for people with musculoskeletal conditions (largely various types of arthritis); however, half used quantitative methods, which may have restricted the richness of information elicited from participants. The barriers and facilitators reported in this rapid review were similar to many of the prominent ones identified in the present study providing further evidence of their key significance, including: prohibitive pain intensity and activity-induced exacerbation of pain (physical capability); lack of knowledge about the health benefits and analgesic effects of PA (psychological capability); fear of causing further harm and the unpredictability of pain symptoms preventing habit formation (automatic motivation); negative beliefs and experiences of PA on pain symptoms (reflective motivation); inaccessible facilities/activities and deterred by lack of time or bad weather (physical opportunity); and lack of company when performing PA (social opportunity).

However, the present study identified a greater range of patient-reported barriers than have been identified in previous studies, including, for example: weight problems making PA difficult; sedating side effects of medication; and fear of the judgement of others. Whilst the rapid umbrella review encompassed current available literature on this topic, the reported findings may have been limited by the inclusion of a high proportion of systematic reviews that included randomised controlled trials predominantly or entirely [32–36]. In consequence, the findings were limited by the data collection methods, which lacked the dynamic interactions and in-depth exploration of topics that can be achieved during qualitative studies. An additional factor that may have limited study findings was the inclusion of numerous studies that focused on barriers to and facilitators of planned and structured exercise, often with a focus on explaining non-adherence to PA interventions [32, 34–39]. The nature of these studies’ objectives means that they are unlikely to have fully captured data concerning general lifestyle PA. Identifying an exhaustive range of barriers is important when considering developing inclusive interventions that have the capacity to anticipate and address the needs of all patients, reduce inequalities and support behavioural change. Study design and method are important considerations in achieving this aim, and the findings of the present study suggest that in-depth qualitative interviews can broaden our understanding of these barriers and facilitators, compared with the data collection methods used in quantitative studies.

The inclusion of stakeholder data in the present study elucidated further, describing barriers that had not been identified by patients. For example, whilst it is often presupposed that healthcare providers will assume responsibility for PA promotion, stakeholders also face barriers related to psychological capability (e.g. lack of knowledge about PA and lack of knowledge around who should assume which responsibilities), which are barriers that were also identified in a recent narrative review [40]. Stakeholders also reported barriers relating to physical opportunity that were faced by patients due to limitations in healthcare systems (e.g. excessive waiting times reducing access to physiotherapy and other healthcare services), and they identified several barriers relating to social opportunity concerning interactions with patients (e.g. lack of their own time to engage with patients and lack of sensitivity and understanding from professionals); limitations in healthcare systems (gaps in service provision and lack of support from specialist services); and PA activities not being the social norm for some patients. These findings suggest that, in aiming to compile as exhaustive an inventory as possible regarding the barriers to and facilitators of PA in people with CP, the inclusion of stakeholder perspectives is imperative. The present study found that these perspectives elucidated the barriers reported by patients, both those that are reported here and those reported in the recent umbrella review [26], which encompasses the current literature on this topic. Additionally, the stakeholder perspectives captured important detail concerning the barriers and limitations encountered by healthcare systems and healthcare providers in aiming to promote PA in this population.

## Implications for research and practice

The identification of important factors in all three constructs of the COM-B model suggests that behavioural change interventions must address multiple aspects. Whilst addressing one component of COM-B has the potential to impact positively on the others, it is likely that maximum effect would be achieved by increasing all three: capability; opportunity; and motivation. The findings of the present study suggest that consideration of these three constructs, and precisely identifying those relevant to individuals, could assist healthcare practitioners in developing a more tailored approach to assessing the barriers encountered by individuals and promoting PA engagement and increased activity levels in people with CP.

### Strengths and limitations

The present study examined both barriers to and facilitators of PA in a sizeable sample of people living with clinically significant CP, with aim of curating as comprehensive a list of barriers and facilitators as possible. A comprehensive list of these factors is the key first step in developing interventions designed to promote PA engagement or to increase activity levels in those already engaging with PA. Researcher preconceptions can result in the potential for bias; however, to mitigate this, an interdisciplinary team was established, and team members were intensively involved in developing the analytical framework and interpreting the findings both inductively and deductively.

## Conclusions and future directions

The barriers to and facilitators of PA in people with CP are numerous and complex, and the inclusion of stakeholder perspectives can broaden our understanding of these factors. Future analyses are planned to investigate the degree of convergence and divergence between patient and stakeholder perspectives, which could further our understanding of potential mismatches in these perspectives and agendas. The COM-B framework provides a mechanism for organising and understanding these barriers and facilitators in a way could that inform intervention design. Future research should develop and evaluate such interventions with the aim of promoting sustainable PA engagement in people with CP. Future directions for the present study include building on this comprehensive catalogue of barriers and facilitators to produce a ‘SUstainable Self Effective Exercise Development’ (SUSSED) intervention to support clinical decision-making. This will involve co-production of the intervention through collaborative efforts between the multidisciplinary research team, people with lived experience of CP and key stakeholders involved in directing policy and practice and in delivering PA support to people living with CP. Michie and colleagues [41] have produced additional guidance, which can be used to develop interventions, based on the known barriers and facilitators for any population and classified according to the COM-B constructs. Taking the barriers and facilitators reported here, this resource could inform the development of interventions that, using the BCW, could support behaviour change through implementation in one or more domains within society (e.g. policy development, service planning or service delivery). Our future work aims to support people living with CP, at the point of service delivery, to engage with PA.

## Supplementary Information

Supplementary Material 1.

Supplementary Material 2.

## Data Availability

The datasets used and/or analysed during the current study are available from one of the senior authors on reasonable request.

## Abbreviations

CP: Chronic pain
CSO: Chief Scientist Office
NHS: National Health Service
PA: Physical activity
