# Supplementary material for: Perceived barriers to and facilitators of physical activity, using the COM-B model for behavioural change, in people with chronic pain: a qualitative evaluation of patient and stakeholder perspectives
Source: BMC Public Health. 2025 Nov 7;25:3859. doi: 10.1186/s12889-025-25252-0 (PMC12595877; doi:10.1186/s12889-025-25252-0)
Supplement: Supplementary file 1 — Supplementary Material 1. [file 12889_2025_25252_MOESM1_ESM.docx]

| **COM-B domain** | **COM-B construct** | **Examples of interview questions/prompts** |
| --- | --- | --- |
| Physical:  Factors that potentially impact on, and are modified by ability to exercise (e.g. disability, BMI, muscle strength, gait, pain characteristics) | Capability | How do you feel about exercising?  What do you know about the role of exercise in managing chronic pain/strengthening your body? |
|  | Opportunity | Tell me about any support or advice you have received about the role of exercise in managing chronic pain?  (Prompts: from doctor, pain clinic staff, physiotherapist, family, friends).  If yes, what was the advice and how did it influence you?  What do your friends and family think about you exercising? |
|  | Motivation | What is your view on the role of exercise in improving your chronic pain/weight/strength? |
|  |  |  |
| Social and physical:  Factors influencing individuals' PA access (e.g. deprivation, family support/ barriers, cost and complexity of available exercise forms, culture, geography, housing, neighbourhood, access to facilities and means to exercise, transport) | Capability | Do you feel you know enough about what exercise you can do to maintain your health and how to plan this into your daily life? If so, how. If not, what is stopping you?  Do you feel you know how to get started on an exercise regimen? If so, how. If not, what is stopping you?  Do you feel confident to plan exercise? If so, how. If not, what is stopping you?  Do you feel that you can overcome obstacles preventing you from exercising posed by social distancing requirements? |
|  | Opportunity | Do you feel supported/confident to engage in exercise within your community/locality? If so, how. If not, what is stopping you? |
|  | Motivation | What do you think is easier – going to an exercise class or exercising on your own?  Is there anything that might get in the way of you engaging in regular exercise? If so, what? |
|  |  |  |
| Reflective:  Willingness to plan and evaluate increased activity, beliefs around chronic pain and activity as treatment | Capability | Do you know how exercise can help you live better with chronic pain? If so, what? |
|  | Opportunity | What things have influenced you to exercise in the past?  Has the requirement for social distancing had any impact on the way you currently exercise? |
|  | Motivation | What do you think exercise can do for keeping you active and healthy? |
|  |  |  |
| Psychological and social:  Current medication (drug burden, particularly opioids) | Capability | Do you feel you know enough about how to manage your pain medication? If yes, what helps you manage your medication, if no, what else do you need to know to better manage your medication?  How confident do you feel to manage your pain with medication? |
|  | Opportunity | Have you been provided with specific advice or help about how to control your pain using medication? |
|  | Motivation | Is there anything you are not taking/doing now which you think might help you manage your pain? |
|  |  |  |
| Psychological:  Fear avoidance, treatment expectation, catastrophizing, self-efficacy, mood, education, knowledge of pain self-management, capacity to engage, ability to plan and carry out instructions | Capability | How do you know how much exercise is appropriate for you? |
|  | Opportunity | Have you been provided with specific advice or help about managing pain-related distress or upset? |
|  | Motivation | What do you think about having exercise recommended for you as a treatment for chronic pain?  How confident do you feel that you can exercise on a regular basis? What increases or decreases your level of confidence? |
|  | | |
| Reflective, social and autonomic:  Reflective factors (outcome expectancies, goals and health), social factors(family and peer support and social contact) and automatic factors (fear of movement, emotional responses, impulsiveness) | Capability | What kind of things influence whether you exercise or not?  Probe: emotion, social, behavioural, cognitive. |
|  | Opportunity | Are there any issues related to exercising that you need more information on? |
|  | Motivation | Exercise is recommended as a good way to keep active with chronic pain. What do think about this recommendation? |
