# Supplementary material for: Perceived barriers to and facilitators of physical activity, using the COM-B model for behavioural change, in people with chronic pain: a qualitative evaluation of patient and stakeholder perspectives
Source: BMC Public Health. 2025 Nov 7;25:3859. doi: 10.1186/s12889-025-25252-0 (PMC12595877; doi:10.1186/s12889-025-25252-0)
Supplement: Supplementary file 2 — Supplementary Material 2. [file 12889_2025_25252_MOESM2_ESM.docx]

| **COM-B domain** | **COM-B construct** | **Examples of interview questions/prompts** |
| --- | --- | --- |
| Physical:  Tailored resources; choice; access; guidelines; education; evidence; signposting; time; safety; instruction; feedback; negotiation; reinforcement/incentives; memory/attention; | Capability | What strategies/initiatives have you noticed are useful to promote exercise in patients with chronic pain?  When is the last time you remember having a conversation with someone suffering chronic pain about exercise? How did that conversation go? How was it received by the patient? |
|  | Opportunity | When you want to recommend/prescribe a particular exercise or activity for someone with chronic pain, how often have you been able to access this?  What barriers exist to accessing hospital based activity/movement advice for patients with chronic pain?  What barriers exist to accessing community based activity/movement advice for patients with chronic pain? |
|  | Motivation | Do you promote exercise regularly or rarely in this group?  To what extent is promoting exercise in those with chronic pain a priority for you? |
|  |  |  |
| Psychological:  Managing resistance; attitudes/beliefs; identity; illness perceptions; confidence to deliver; perceptions of ability; perceptions of success and impact; | Capability | What do you think about exercise as a way to manage chronic pain?  How easy do you think it is for people with chronic pain to exercise? Prompt: tell me more about why it is easy or difficult?  What impact do you think discussing this would have? |
|  | Opportunity | How much support is there for those with chronic pain to engage in exercise? |
|  | Motivation | What do you think are the benefits of exercise for those with chronic pain? |
|  |  |  |
| Social:  Communication; language; trust; fear appeals | Capability | What type of exercise do you think would be helpful for people with chronic pain?  Have you ever recommended exercise and were unsure of its suitability for someone with chronic pain? |
|  | Opportunity | How available are opportunities for those with chronic pain to participate in exercise in the community?  What impact has social distancing had on those opportunities? |
|  | Motivation | What do you think are the harms of those with chronic pain exercising?  What do you think are the concerns that those with chronic pain have regarding exercise? |
